# Exceptionally rapid tooth development and ontogenetic changes in the feeding apparatus of the Komodo dragon

**DOI:** 10.1371/journal.pone.0295002

**Published:** 2024-02-07

**Authors:** Tea Maho, Robert R. Reisz

**Affiliations:** 1 International Center of Future Science, Jilin University, Changchun, Jilin Province, China; 2 Department of Biology, University of Toronto Mississauga, Mississauga, Ontario, Canada; State Museum of Natural History, GERMANY

## Abstract

Dental developmental and replacement patterns in extinct amniotes have attracted a lot of attention. Notable among these are Paleozoic predatory synapsids, but also Mesozoic theropod dinosaurs, well known for having true ziphodonty, strongly serrated carinae with dentine cores within an enamel cap. The Komodo dragon, *Varanus komodoensis*, is the only extant terrestrial vertebrate to exhibit true ziphodonty, making it an ideal model organism for gaining new insights into the life history and feeding behaviours of theropod dinosaurs and early synapsids. We undertook a comparative dental histological analysis of this extant apex predator in combination with computed tomography of intact skulls. This study allowed us to reconstruct the dental morphology, ontogeny, and replacement patterns in the largest living lizard with known feeding behaviour, and apply our findings to extinct taxa where the behaviour is largely unknown. We discovered through computed tomography that *V*. *komodoensis* maintains up to five replacement teeth per tooth position, while histological analysis showed an exceptionally rapid formation of new teeth, every 40 days. Additionally, a dramatic ontogenetic shift in the dental morphology of *V*. *komodoensis* was also discovered, likely related to changes in feeding preferences and habitat. The juveniles have fewer dental specializations, lack true ziphodonty, are arboreal and feed mostly on insects, whereas the adults have strongly developed ziphodonty and are terrestrial apex predators with defleshing feeding behaviour. In addition, we found evidence that the ziphodont teeth of *V*. *komodoensis* have true ampullae (interdental folds for strengthening the serrations), similar to those found only in theropod dinosaurs. Comparisons with other species of *Varanus* and successive outgroup taxa reveal a complex pattern of dental features and adaptations, including the evolution of snake-like tongue flicking used for foraging for prey. However, only the Komodo dragon exhibits this remarkable set of dental innovations and specializations among squamates.

## Introduction

*Varanus komodoensis* (Komodo dragon), restricted to four islands in Indonesia, is not only the largest extant lizard but is also listed as endangered. Their unique position among extant lizards and their position as apex predators on these islands has attracted a lot of attention, and their feeding behaviour has been extensively studied through direct observations of movement [1], examination of the tooth structure [2], the biting and pulling forces [3], and identification of the tooth marks found on the prey bone surface [4].

Members of the genus *Varanus* frequently experience an ontogenetic shift in their diets to maximize the available food resources as they grow [5]. This shift has been well documented in *V*. *komodoensis*, with juveniles being arboreal (approximately 8 months) and transitioning to terrestrial habitats as adults, their food source changing from insects and lizards to larger mammals [1,6]. Although this shift in diet is quite dramatic for *V*. *komodoensis*, previous research has suggested that there is almost no ontogenetic change in tooth morphology [1], generally described as laterally compressed, serrated on the cutting edges of the crown, and pleurodont implantation [2,7–9].

An interesting dental characteristic noted for *Varanus komodoensis* is the presence of serrations (denticles) along the carinae [4]. Such serrations in terrestrial predators can be subdivided into two categories, small serrations in various carnivorous predatory vertebrates resulting from variations in the thickness of the enamel layer of the tooth [10] that may be readily worn down, and more prominent permanent serrations that are supported by dentine cores and are designated as ziphodont [4,11,12]. Among extinct vertebrates, the former is present in the sabre-toothed mammal, *Smilodon fatalis* [13], while the latter has been shown to be present in some species of the early Permian sphenacodontid synapsid *Dimetrodon* [10] and most theropod dinosaurs [12,13]. However, little is known about these types of serration morphology in members of *Varanus*, the most spectacular extant lizard predators.

Numerous extant squamates replace their teeth continuously (polyphyodonty) [14] throughout their lifetime, as is also the case in extant *Alligator* and *Caiman* [15], extinct synapsids [16], and dinosaurs [17,18]. Replacement occurs when a more recently developed tooth comes to replace the functional tooth in a particular position on the jaw. The rate at which the process of replacement occurs has been of great interest because of its effect on feeding strategies and overall behaviour [15,19,20]. The age of a tooth is measured by counting the daily formation of von Ebner lines within the dentin, well documented in various taxa, including crocodilians and dinosaurs [15,20]. The rate of replacement is measured here by the replacement rate or how often (measured in days) a new tooth begins to form.

The pattern of tooth development and ontogeny is governed by several variables in polyphyodont vertebrates, but the interaction of these variables has yet to be studied in any detail. For example, previous research reported that *Varanus flavescens* and *Varanus salvator* had one replacement tooth per position, while *V*. *komodoensis* had multiple teeth lingual to the functional tooth [14,21]. Thus, a rapid tooth replacement rate has been suggested for *V*. *komodoensis* but not quantitatively evaluated. A detailed comparative study of the dentition of the Komodo dragon is particularly important since this endangered varanid is one of the longest-lived species of *Varanus*, with some individuals reaching 60 years of age [22,23], and its dental morphology can be used as analogues for carnivorous early synapsids and dinosaurs to gain a better understanding of their feeding behaviours.

Previous studies have focused on the unique feeding behaviour of the Komodo dragon but have not related this to dental morphology, development, and replacement. To gain a better understanding of the unique set of behaviours exhibited by the Komodo dragon, we examine for the first time the dentition and jaw elements of adult and juvenile *V*. *komodoensis* with a combination of skeletonized specimens and shed teeth for histological analysis and computed tomography (CT) of whole skulls. Our surprising discoveries led us to place our findings in a broader perspective through comparisons with other species of *Varanus* and to three outgroup taxa, *Lanthanotus borneensis*, *Heloderma suspectum*, and *Shinisaurus crocodilurus*, sequentially more distant relatives of this genus [24]. Since varanids were previously considered snake relatives [25–27], we also included two booid snakes in our comparisons. This combined approach provides a more complete understanding of the variables that define the patterns of tooth development, replacement, and longevity in the Komodo dragon and their relevance to the feeding behaviour of this apex predator.

## Methods and materials

Skulls located in the collections of the Royal Ontario Museum (ROM) for *Varanus komodoensis* (ROM R8973) from the Toronto Zoo, *Varanus exanthematicus* (ROM R8034) from Little Ray’s Nature Centre in Ottawa, *Varanus salvator* (ROM R8972) from Malaysia, *Varanus salvadorii* (ROM R9105) from Papua New Guinea, *Python molurus bivittatus* (ROM R9169) from Little Ray’s Nature Centre in Ottawa, and *Boa constrictor* (ROM R7465) from Alta Vista Animal Hospital in Ottawa, were used to remove a tooth family (functional and replacement tooth) from the jawbone for histological analysis. All of the specimens were photographed labially, lingually, and occlusally using Leica DVM6 digital microscope and LAS X software prior to histological analysis. Terminology for dentition morphology is consistent with Hendrickx et al. [28]. No permits were required for the described study, which complied with all relevant regulations.

### Histology

The functional and replacement teeth for *Varanus komodoensis*, *Varanus salvadorii*, *Varanus salvator*, *Varanus exanthematicus*, *Boa constrictor*, and *Python molurus bivittatus* were individually embedded in Castolite AC polyester resin, vacuumed, and left to cure for a minimum of 24-hours before cutting. The Metcut-5 low-speed saw (MetLab) with a diamond wafer blade (225 rpm) was used to cut the specimens. For the smaller isolated elements (e.g. replacement teeth), the cut was made slightly off to one side to prevent a kerf loss at the apex of the teeth. The specimens were mounted on frosted plexiglass slides and cut again using the Metcut-5 saw to a thickness of approximately 1 mm. The specimens were ground using the Metcut-10 Geo (MetLab) machine with a grinding cup, followed by manual grinding with a progressively finer grit (1000- to 2000-grit) silicon carbide paper. The specimens were imaged using a Nikon DS-Fi1 camera mounted onto a Nikon AZ-100 microscope using NIS Elements-Basic Research software registered to R. R. Reisz of the University of Toronto Mississauga.

### Tooth longevity and replacement rate

The replacement rate and the tooth development time were examined for a tooth family, consisting of the functional tooth and its successive replacement teeth that are developing on the lingual side of the jawbone [14,17]. The incremental lines of von Ebner were counted for each tooth within a family (functional and replacement teeth) to calculate the replacement rate, which was determined by subtracting the total incremental lines of von Ebner of the replacement tooth from the total incremental lines of von Ebner of the functional tooth. This method was repeated for the tooth families which had multiple replacement teeth per tooth position, so the total lines of the second replacement tooth was subtracted from the total lines of first replacement tooth. For the thin sections, the counts for the incremental lines were made starting at the pulp cavity and continuing to the exterior edge of the tooth and continuing towards the tooth apex. When not all the lines were present within a given area, the average width of the incremental line was used for the width of that specific dentine area, thus the method described by Maho et al. (2022) was used to estimate the number of missing lines within that area [16]. For the teeth where no incremental lines of von Ebner were visible, the mean line width from either the functional or the replacement tooth was used to estimate the missing lines of the other tooth within the tooth family. This method was used for the first replacement tooth of *V*. *salvator* since no lines were present throughout the entire dentine area.

### Computed Tomography (CT) images

The data contains computed tomography (CT) scans from MorphoSource of six skulls for the *Varanus* taxa: adult *V*. *komodoensis*, juvenile *V*. *komodoensis*, *V*. *salvator*, *V*. *exanthematicus*, *V*. *acanthurus*, and *V*. *gouldii*. All of the specimens were scanned at the University of Texas High-resolution X-ray computed tomography (HRXCT) Facility. The *V*. *komodoensis* adult (TNHC 95803) was scanned at 150kV with a total of 1,816 equally-spaced TIFF slices with a voxel size of 0.1626 × 0.1626 × 0.1626 mm [29]; while the juvenile (TNHC 102417) was scanned at 140kV with a total 1,942 equally-spaced TIFF slices with a voxel size of 0.0274 × 0.0274 × 0.0274 mm. *Varanus salvator* (FMNH 35144) was scanned at 180kV with a total of 750 TIFF slices with a voxel size of 0.088 x 0.088 x 0.201mm. *Varanus exanthematicus* (FMNH 58299) was scanned at 180kV, 0.1333 mA, no filter, with a total of 930 equally-spaced TIFF slices with a voxel size of 0.05273 x 0.05273 x 0.11855 mm. *Varanus acanthurus* (UTA 13015) was scanned at 180kV, 0.1333 mA, no filter, with a total of 1140 equally-spaced TIFF slices with a voxel size of 0.02328 x 0.02328 x 0.05118 mm. Lastly, *Varanus gouldii* skull (TMM M-1295) was scanned at 150 kV, 0.16 mA, no filter, with a total of 384 equally-spaced TIFF slices with a voxel size of 0.084 x 0.084 x 0.2095 mm.

Additionally, CT data for three outgroups were examined: *Lanthanotus borneensis* (FMNH 148589), was scanned at 180kV with a total of 660 TIFF slices with a voxel size of 0.02148 x 0.02148 x 0.04637 mm. *Heloderma suspectum* (TNHC 62766) was scanned at 180kV with a total of 555 TIFF slices with a voxel size of 0.0542 x 0.0542 x 0.133 mm. *Shinisaurus crocodilurus* (TNHC 62987) was scanned at 180kV with a total of 465 TIFF slices with a voxel size of 0.0293 x 0.0293 x 0.0784 mm.

Using ImageJ, version 1.53a, all of the 16-bit TIFF slices for the *Varanus* skulls were individually converted to 8-bit and stacked in an image sequence. The specimens were then rendered and segmented on Avizo Lite, version 2020.3, registered to R. R. Reisz at the University of Toronto Mississauga.

## Results

### *Varanus komodoensis* dental ontogeny

**Fig 1:**
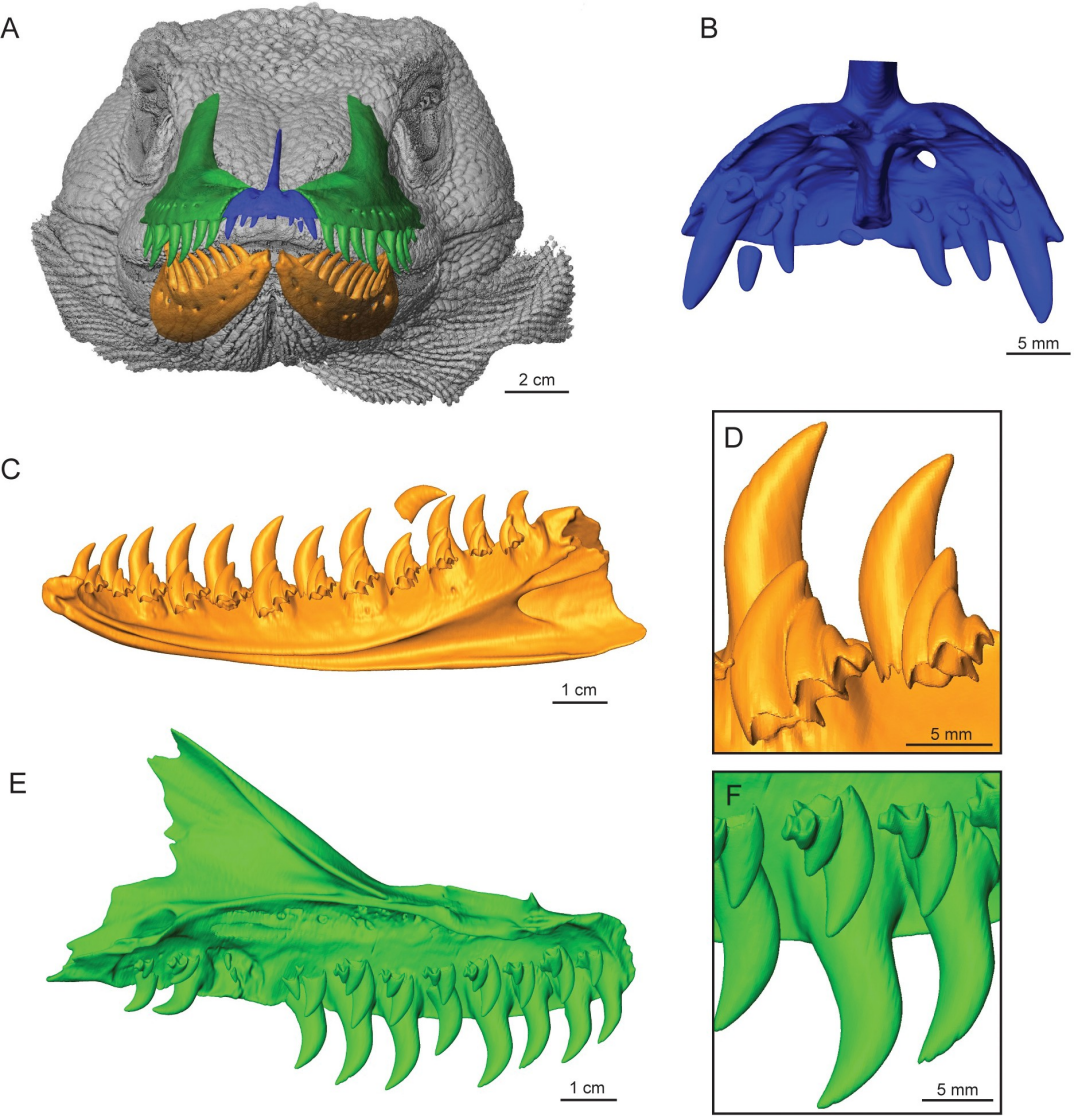
Adult *Varanus komodoensis* jaw renderings. (A) TNHC 95803, isosurface rendering of complete specimen showing the relative position of jaw elements. (B) Premaxilla, lingual view. (C) Right dentary, lingual view. (D) Close-up of tooth positions 6 and 7 of the dentary. (E) Left maxilla, lingual view. (F) Close-up of tooth positions 3 and 4 of the maxilla.

**Fig 2:**
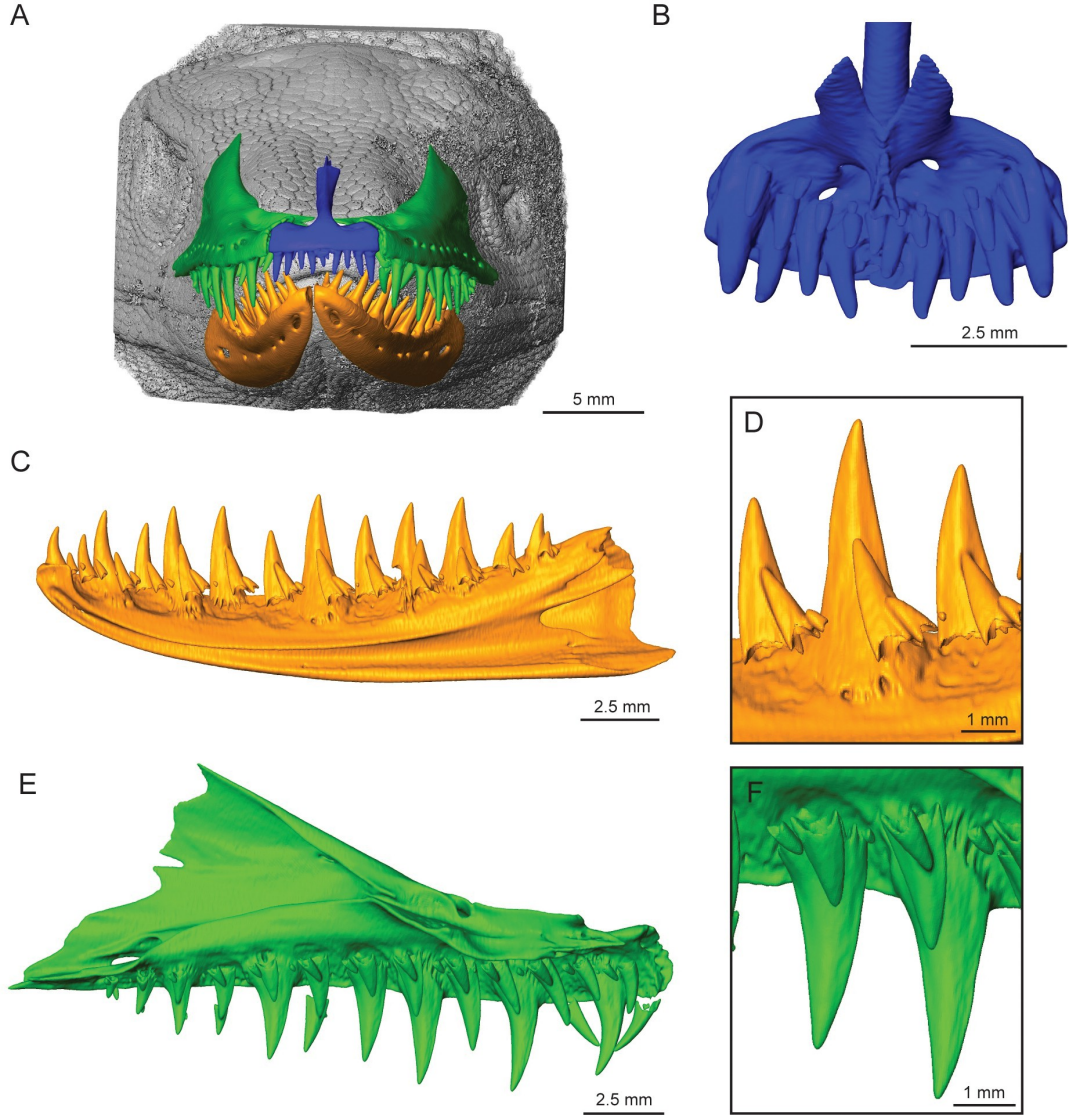
Juvenile *Varanus komodoensis* jaw renderings. (A) TNHC 102417, isosurface rendering of complete specimen showing the relative position of jaw elements. (B) Premaxilla, lingual view. (C) Right dentary, lingual view. (D) Close-up of tooth positions 7 to 9 of the dentary. (E) Left maxilla, lingual view. (F) Close-up of tooth positions 6 and 7 of the maxilla.

Direct examination and CT data show that the adult *Varanus komodoensis* (TNHC 95803) teeth are indeed strongly labio-lingually compressed on both the maxilla and dentary, with wide bases and become narrower towards the crown apex. There is strong recurvature throughout the entire tooth row, with curvature increasing more posteriorly as tooth height decreases (Fig 1A–1E). Both juvenile *V*. *komodoensis* skulls, TNHC 102417 and ROM R8973, teeth have labio-lingual compression of the maxillary and dentary teeth, but differences in tooth shape in the three *V*. *komodoensis* individuals illustrate the ontogenetic shift in dental morphology. In contrast to the adult condition, there is minimal recurvature in TNHC 102417, mainly observed in the anterior jaw region, while the other teeth have simple, sharply pointed crowns (Fig 2A–2E), while ROM R8973 appears to be at a slightly later ontogenetic stage than TNHC 102417 since the overall length of the maxilla is greater, and the teeth are more recurved.

The premaxillary (pmx) teeth curve inward (lingually) and are smaller in size than the dentary and maxillary teeth, with the smallest being at the midline (pmx1 and pmx2). For the adult, the tooth height difference between pmx3 and pmx5 is double, while the juvenile appears to have a similar height for all premaxillary teeth (S1 Table). The anterior teeth are smaller in size for both the maxilla and dentary, with tooth size slightly increasing posteriorly to a consistent height for the middle section and decreasing for the most posterior teeth. The first maxillary tooth (mx1) is larger than the last premaxillary tooth (pmx5) in the adult, but this could not be compared with the juvenile condition since only small replacement teeth were present in the same tooth position.

**Fig 3:**
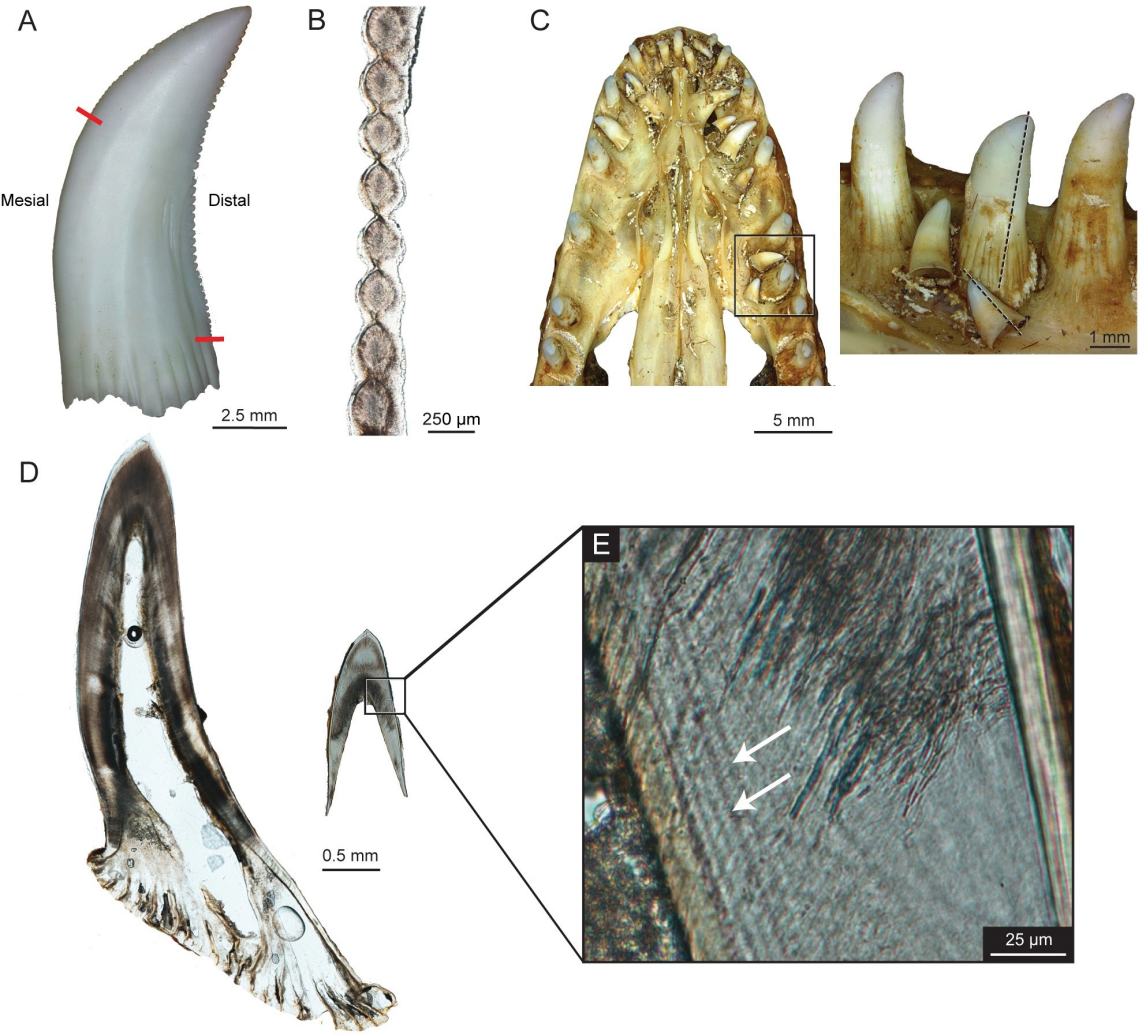
Histological thin sections for *Varanus komodoensis*. (A) ROM R10117, shed tooth showing mesial and distal serrations with red lines illustrating the end of the ziphodonty. (B) ROM R10117, distal ziphodonty. (C) ROM R8973, occlusal view of the skull roof and lingual view of tooth position 6, with dashed black lines representing the thin section. (D) Complete longitudinal thin section for the functional and replacement tooth. (E) Close-up section of replacement tooth showing incremental lines of von Ebner with white arrows.

**Fig 4:**
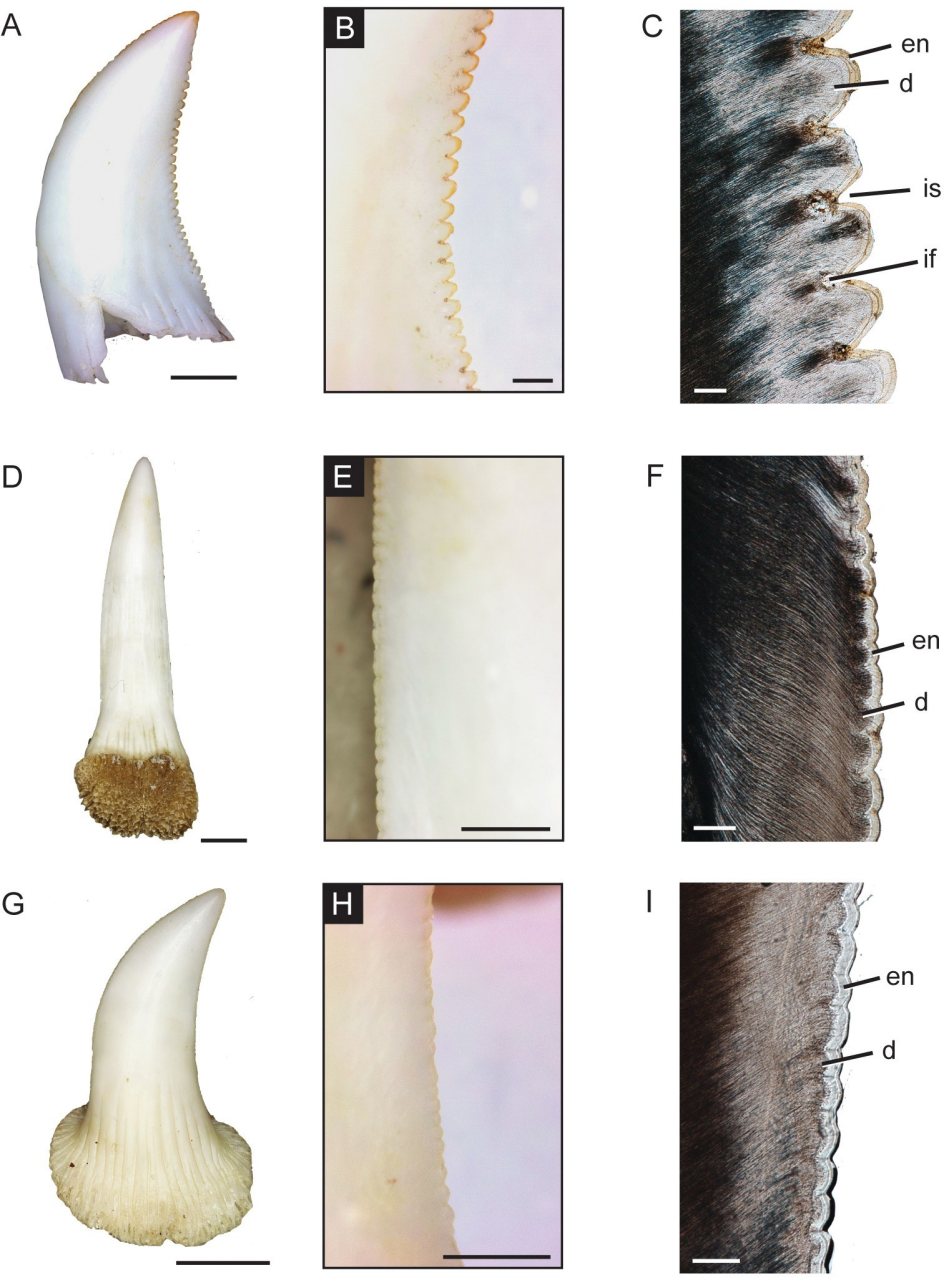
Tooth crown serrations of *Varanus* taxa. (A) Adult *V*. *komodoensis* (ROM R10163) shed tooth. (B) Close-up of distal ziphodonty. (C) Cross-section of ziphodonty. (D) *V*. *salvadorii* tooth, labial view (ROM R9102). (E) Close-up of mesial serrations. (F) Cross-section of ziphodonty. (G) *V*. *salvator* tooth, lingual view (ROM R8972). (h) Close-up of distal serrations. (I) Cross-section of ziphodonty. Abbreviations: d, dentine; en, enamel; if, interdental fold; is, interdental sulcus. a, d, g scale = 2 mm; b, e, h scale = 500 μm; and c, f, i scale = 100 μm.

**Fig 5:**
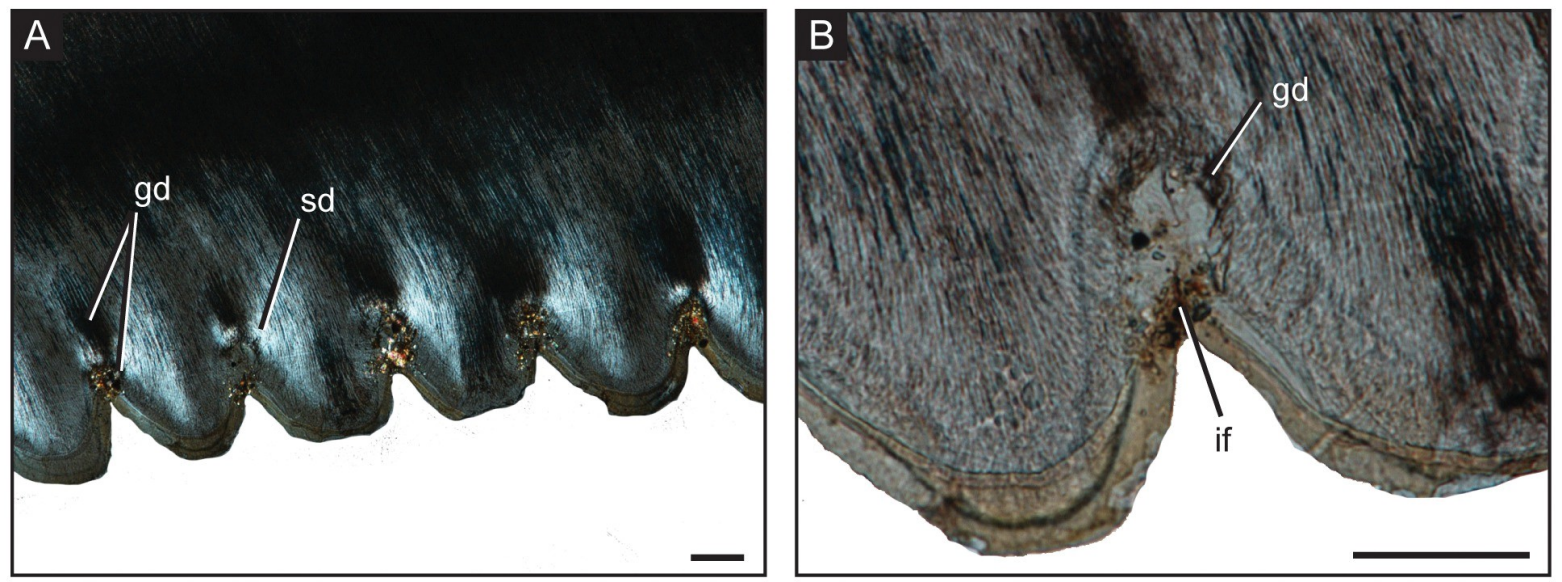
Microanatomy of *Varanus komodoensis* interdental folds. (A) Antero-posterior longitudinal thin section showing ziphodonty on distal crown edge, under cross-polarized light. (B) Close-up of the interdental fold (ampulla) between adjacent denticles. Abbreviations: gd, globular dentine; if, interdental fold; sd, sclerotic dentine. Scale = 100 μm.

The adult *V*. *komodoensis* exhibits true ziphodonty, the serrations on the carinae being composed of both the enamel cap and a dentine core, rather than just enamel [10] on both the mesial and distal surfaces (Figs 3A–3C and 4A–4E). On the mesial side, ziphodonty appears only on the top third of the crown, whereas the distal ziphodonty extends throughout the entire crown edge (Fig 3A), extending slightly past the external plicidentine where the serrations also appear to flare out distally, and moving further basally the smaller-sized serrations become slightly positioned more labially. A greater number of serrations were present on the distal edge compared to the mesial edge, 43 and 23, respectively. The distal serrations exhibit deeper interdental sulci that make the external surface more prominent, as shown by the greater average length and height of the individual serrations (Table 1 and Fig 4B). Similar to theropod dinosaur serrations, the interdental sulci allow for the denticles to have a greater height within the dentine [12]. The serrations are smaller apically, increase near the mid-crown, and decrease in size basally (Fig 3A). The dentinal cores surrounded by the enamel were visible through the histological thin sections (Figs 3B and 4C). On the distal carinae, ampullae (interdental folds [12]), similar to those seen in theropod dinosaurs, were observed between adjacent denticles with cracks found in the enamel (Fig 4C). These appeared as hollow, circular spaces within the antero-posterior longitudinal cross-section (Fig 5A and 5B), surrounded by globular dentine, which is dark in appearance and sclerotic dentine, which is transparent [12]. Overall, the serrations are larger, and there was a greater number of individual serrations on both edges in the adult teeth than in the juvenile. The serrations were not well-developed in the juvenile, did not have dentine cores, and thus not ziphodont. The juvenile dentition has more serrations on the distal side, which appear larger near the mid-crown and either reduced or completely absent on the crown apex and base (Table 1).

**Table 1:** Morphological measurements of serrations. Amount and total length of serrations on the tooth crown for *V*. *komodoensis*, *V*. *salvadorii*, and *V*. *salvator*.

Specimen	Number of serrations		Serration length (mm)		Tooth height of labial side (mm)
	Distal	Mesial	Distal	Mesial	
*V*. *komodoensis* (ROM R10117)	43	23	11.28	6.16	14.28
*V*. *komodoensis* (ROM R8973)	24	Not clearly defined	2.37	1.95	3.47
*V*. *salvadorii* (ROM R9102)	123	115	11.33	11.59	13.12
*V*. *salvator* (ROM R8972)	45	46	3.72	3.79	4.65

### *Varanus komodoensis* tooth replacement

CT data of TNHC 95803 showed the presence of two to five replacement teeth for each tooth position in both the dentary and the maxilla (Fig 1C–1F). Detailed examination indicates that seven out of the twelve tooth positions on the right dentary had four to five replacement teeth (Fig 1C). In the few instances that the fifth replacement tooth was identified, it was only visible through the individual cross-sections of the CT data since the tooth was in the very early stages of development. Unlike the adult *V*. *komodoensis*, the juvenile had two to three replacement teeth per tooth position (Fig 2B–2F).

A tooth family, including the functional and replacement tooth, in the juvenile was sectioned for a left maxilla at position 6 (ROM R8973). The functional tooth had approximately 96 incremental lines, while its replacement had 56 lines, resulting in a very short replacement rate of 40 days (Fig 3D–3E; Table 2). Additionally, replacement occurs in an alternating pattern, where every other tooth is either in the process of replacement or has the same amount of replacement teeth within the same stage of development (Figs 1B–1F and 2B–2F), as Edmund [14] suggested.

**Table 2:** Counts for incremental lines of von Ebner. Total number and mean width of incremental lines of von Ebner in functional tooth (FT) and replacement teeth (RT) and replacement rate (days) for *V*. *komodoensis*, *V*. *salvadorii*, *V*. *salvator*, *V*. *exanthematicus*, *Python molurus bivittatus*, and *Boa constrictor*. “None” means that no tooth was present, while N/A means that the tooth did not exhibit any discrete visible incremental lines of von Ebner.

Specimen	Total number of incremental lines			Replacement rate (days)		Mean incremental line width (μm)		
	FT	RT 1	RT 2	FT–RT 1	RT 1 –RT 2	FT	RT 1	RT 2
*V*. *komodoensis* (ROM R8973)	96	56	None	40	None	5.11 ± 0.26	5.68 ± 0.29	None
*V*. *salvadorii* (ROM R9105)	139	96	49	42	47	6.23 ± 0.52	5.89 ± 0.82	7.22 ± 0.84
*V*. *salvator* (ROM R8972)	122	63	10	59	52	7.73 ± 0.77	N/A	7.15 ± 0.59
*V*. *exanthematicus* (ROM R8034)	173	64	None	109	None	8.77 ± 1.13	7.04 ± 0.32	None
*Python molurus bivittatus* (ROM R9169)	144	51	N/A	93	N/A	3.99 ± 0.87	N/A	N/A
*Boa constrictor* (ROM R7465)	84	26	None	58	None	5.28 ± 0.56	5.15± 0.53	None

### Tooth morphology and replacement in other species of *Varanus*

**Fig 6:**
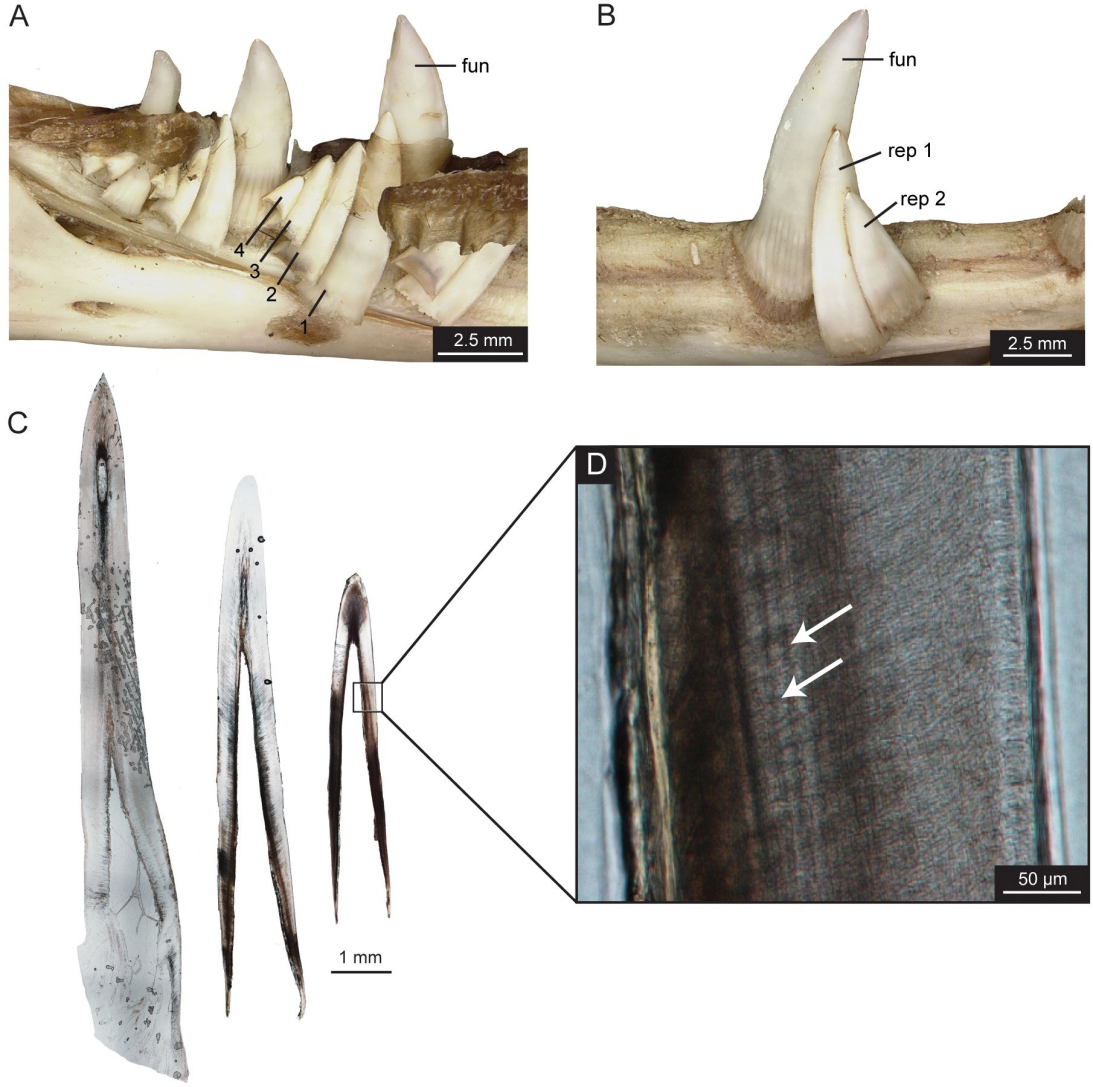
*Varanus salvadorii* jaw and histological sections. (A) Left dentary, ROM R6783, tooth positions 9 and 10 with functional and four replacement teeth, labelled 1 to 4. (B) Right dentary, ROM R9105, tooth position 7 with functional and two replacement teeth. (C) Complete longitudinal thin sections for ROM R9105 functional and two replacement teeth. (D) Close-up section of replacement tooth showing incremental lines of von Ebner with white arrows. Abbreviations: fun, functional tooth; rep 1, replacement tooth 1; rep 2, replacement tooth 2.

The teeth of *Varanus salvadorii*, the closest extant relative of *Varanus komodoensis*, are similarly labio-lingually compressed teeth with small-sized serrations on both the mesial and distal carinae. Unlike *V*. *komodoensis*, the serrations on both tooth edges extend throughout the entire crown length (Fig 4D). The individual serrations are irregularly shaped and more numerous (123 on distal and 115 on mesial) but have a reduced size with a smaller denticle length and shallow interdental sulci (Table 1). The serrations were mostly made up of enamel, with shallow dentine cores observed below, and no ampullae (Fig 4F). The skeletonized skull of the juvenile *V*. *salvadorii* (ROM R6783) had a maximum of four replacement teeth per tooth position (Fig 6A). For the sub-adult dentary (ROM R9105), there were only a maximum of two replacement teeth preserved in position for a few of the tooth families (Fig 6B), most likely the result of the skeletonization process. Within the tooth family, all three teeth showed clear incremental lines of von Ebner but not throughout the entire dentine section (Fig 6C and 6D). The replacement rate for tooth position 7 was determined to be 43 days between the functional and first replacement tooth and 47 days between the first replacement and the second replacement tooth (Table 2). The premaxillary teeth are smallest at the midline and increase in size laterally; however, the last premaxillary tooth is still smaller in height than the first maxillary tooth (Fig 6A).

**Fig 7:**
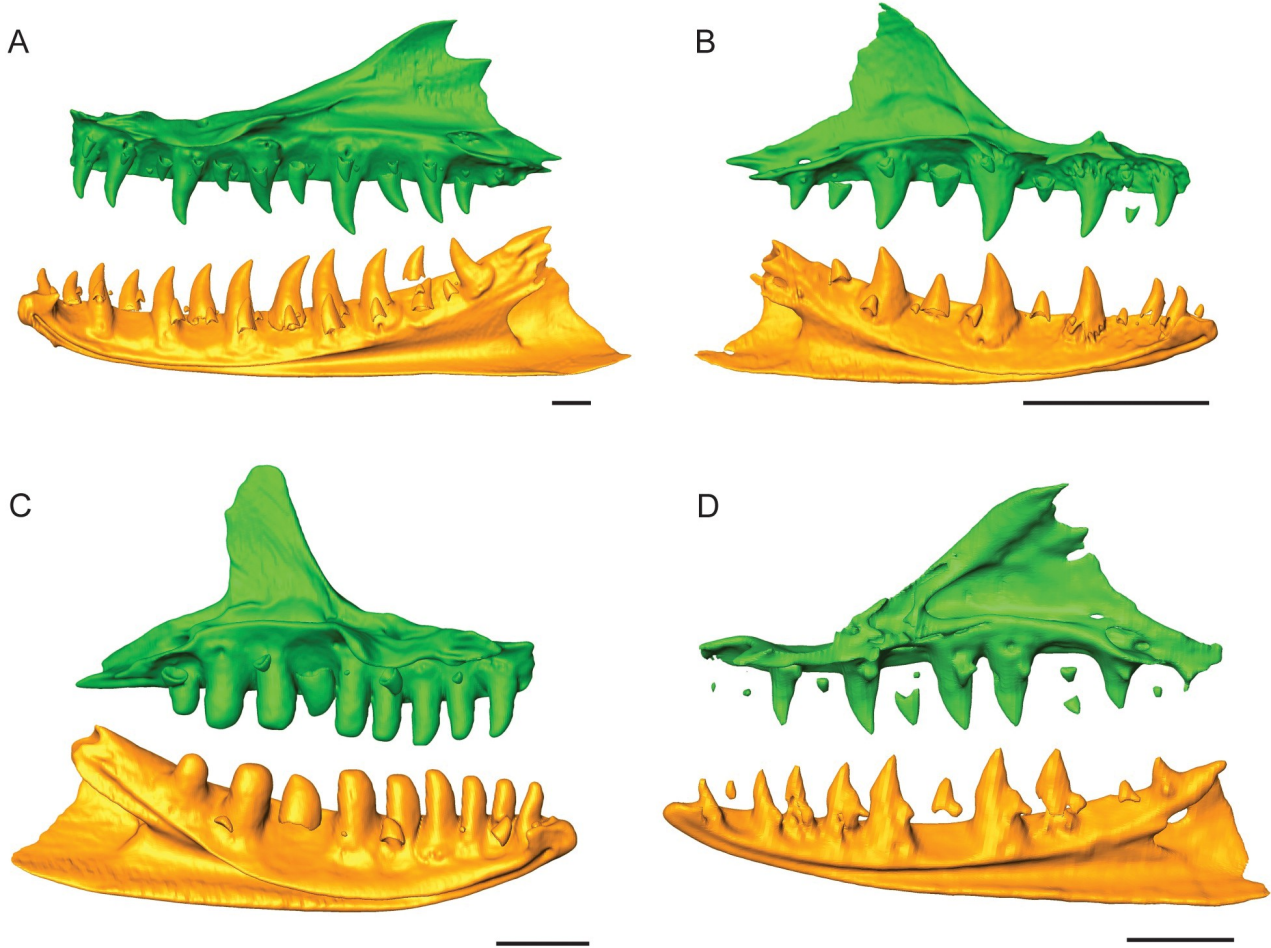
Jaw renderings for other varanid lizards. Lingual view for maxilla (green) and dentary (orange) bones for (A) *Varanus salvator*, FMNH 35144. (B) *Varanus acanthurus*, UTA 13015. (C) *Varanus exanthematicus*, FMNH 58299. (D) *Varanus gouldii*, TMM M-1295.

**Fig 8:**
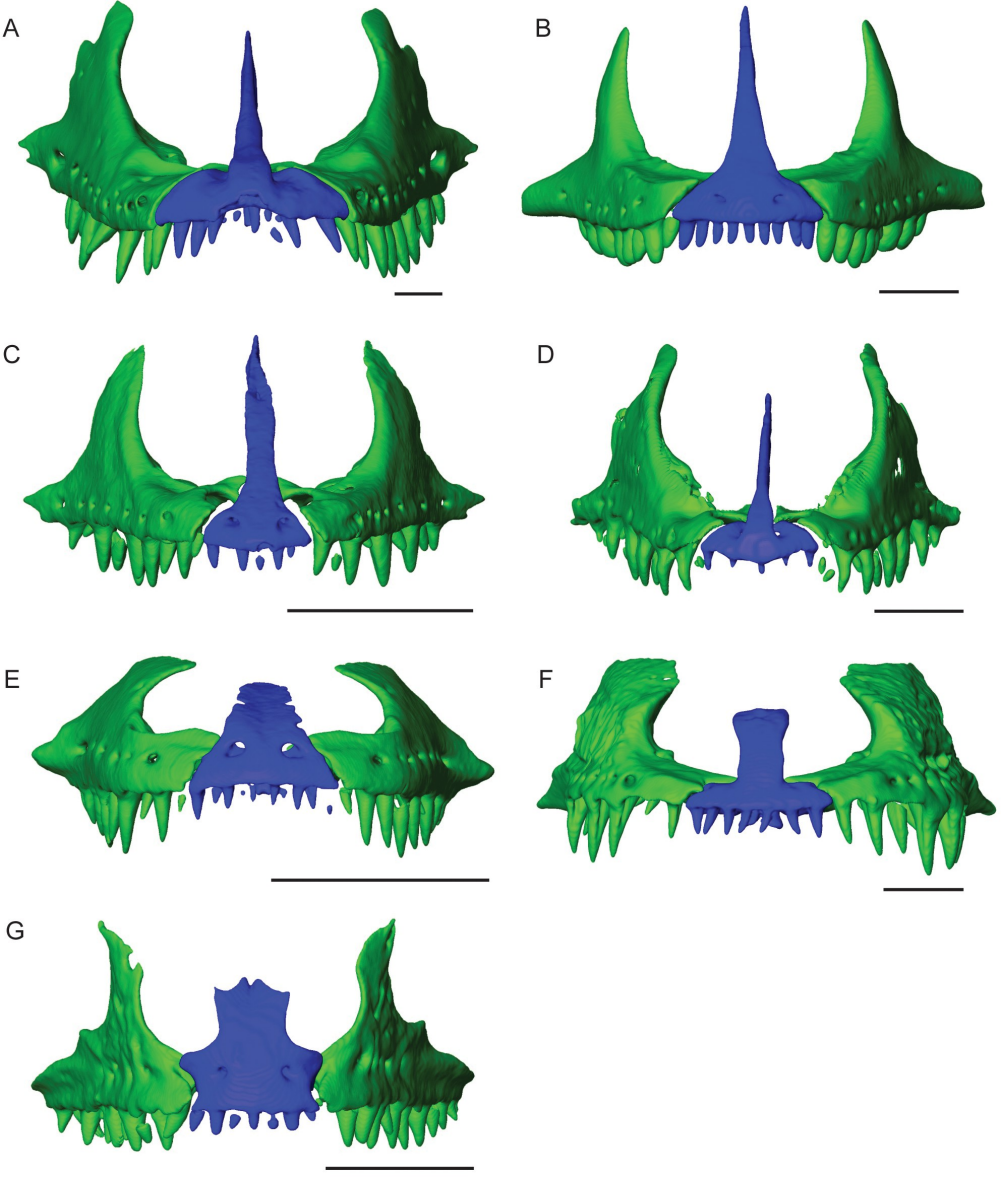
Premaxilla renderings. Anterior view of the jawbones, premaxilla (blue), maxilla (green) and dentary (orange), for (A) *Varanus salvator*, FMNH 35144. (B) *Varanus exanthematicus*, FMNH 58299. (C) *Varanus acanthurus*, UTA 13015. (D) *Varanus gouldii*, TMM M-1295. (E) *Lanthanotus borneensis*, FMNH 148589. (F) *Heloderma suspectum*, TNHC 62766. (G) *Shinisaurus crocodilurus*, TNHC 62987. Scale = 5 mm.

*Varanus salvator* (FMNH 35144) has labio-lingually compressed teeth [21] for both the maxilla and dentary, with recurvature appearing to remain uniform throughout the tooth row (Fig 7A). The irregularly shaped serrations extend mesially and distally on the mid-section of the tooth crown, while absent on the apex (Table 1; Fig 4G–4I). Small incipient dentine cores are present below the thick enamel, where the dentine follows the pattern of the thick enamel, but the enamel-dentine junction does not form distinct folds, and no ampullae are present between adjacent denticles (Fig 4I). The premaxillary dentition is smaller than the marginal dentition, with the smallest teeth occurring in the middle and an increase in tooth height posteriorly (Fig 8A). The first and second premaxillary teeth were missing, or only replacement teeth were in position, so we were not able to calculate total tooth height for these positions. The last premaxillary tooth was slightly smaller than the first maxillary tooth. The CT data reveal a maximum of 2 replacement teeth per tooth position on both the maxilla and dentary (Fig 7A) [14,21]. In addition, the dentary tooth position 7 (ROM R8972) was histologically sectioned and showed a replacement rate of 59 days for the functional and first replacement tooth and 53 days for the first replacement and the second replacement tooth (Table 2).

*Varanus acanthurus* has smaller teeth that still exhibit labio-lingual compression and slight recurvature with a sharp apex (Fig 7B). The bases are expanded with a narrowing towards the apex, and there is an increase in width for the tooth bases posteriorly along the tooth row. *Varanus exanthematicus* has conical teeth that are blunt apically, with fluting around the crown apex and no serrations present on the tooth edges. The anterior teeth are more slender and taper apically with slight recurvature, compared to the posterior teeth, which appear to increase in tooth length and with less tapering at the tooth apex and no recurvature (Fig 7C). *Varanus gouldii* has labio-lingually compressed teeth with expanded bases and slight recurvature (Fig 7D). The CT data for *V*. *exanthematicus (*FMNH 58299), *V*. *acanthurus* (UTA 13015), and *V*. *gouldii* (TMM M-1295) showed smaller premaxillary teeth when compared to the maxilla and only one replacement tooth per tooth position (Fig 8B–8D). Additionally, only the replacement rate for *V*. *exanthematicus* could be determined, with the maxilla tooth position 7 (ROM R8034) being 109 days between the functional and replacement tooth (Table 2).

### Tooth morphology in outgroup taxa

*Lanthanotus borneensis*, generally considered to be the sister taxon to *Varanus*, has labio-lingually compressed teeth with an expanded base, sharp apex, and recurvature [30]. The premaxillary teeth are smaller medially and increase in size laterally towards the maxillary teeth, with the first maxillary tooth being the same height as the last premaxillary tooth (Fig 8E). The CT data show that *L*. *borneensis* has one replacement tooth per tooth position.

In the helodermatid *Heloderma suspectum*, teeth are conical, sharply pointed, with recurvature. For both the maxilla and dentary, the teeth are smaller in tooth height anteriorly, increase in the middle, and decrease in tooth height posteriorly [30]. The majority of the premaxillary teeth are similar in size, with a slight increase in height observed for the last tooth (Fig 8F). The CT data show that *H*. *suspectum* has a maximum of two replacement teeth, similar to *Heloderma horridum*.

*Shinisaurus crocodilurus* has conical teeth with slight narrowing towards the tooth apex, with no recurvature [30]. The dentary and maxillary teeth appear similar in height, except for the first few teeth being slightly smaller. The premaxillary teeth appear to be similar in size (tooth height; S1 Table; Fig 8G). Additionally, *S*. *crocodilurus* had one replacement tooth per tooth position.

**Fig 9:**
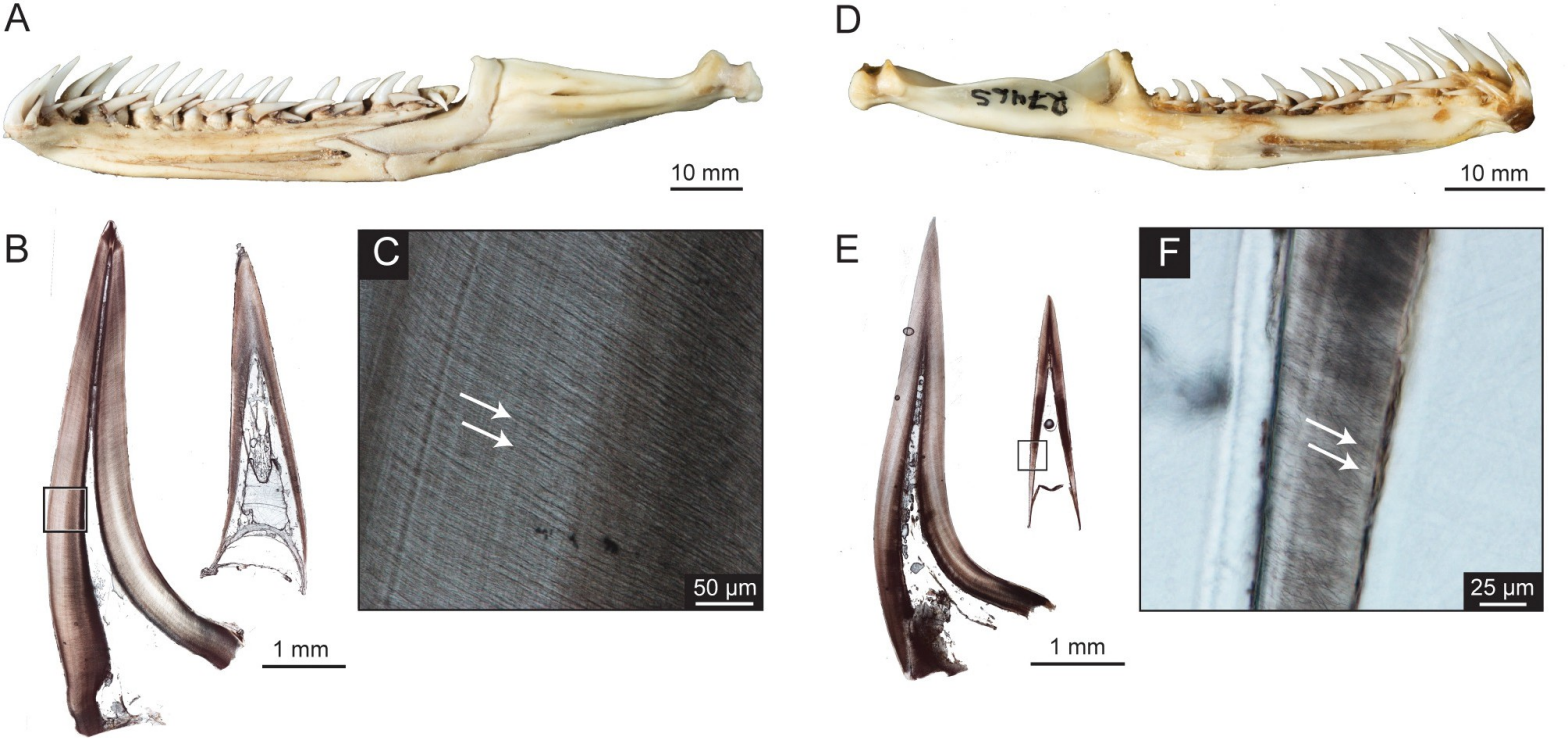
Tooth histological thin sections for snakes. *Python molurus bivittatus* (ROM R9169) (A) Photograph of right dentary. (B) Complete longitudinal thin sections for functional and first replacement tooth. (C) Close-up section of functional tooth. *Boa constrictor* (ROM R7465) (D) Photograph of left dentary. (E) Complete longitudinal thin sections for functional and first replacement tooth. (F) Close-up section of replacement tooth. Incremental lines of von Ebner are shown with white arrows.

The teeth on the left dentary of *Boa constrictor* (ROM R7465) were observed to have approximately two replacement teeth per functional tooth (Fig 9D–9F), while the right dentary of *Python molurus bivittatus* (ROM R9169) had between two to three replacement teeth (Fig 9A–9C), which has been previously noted for booid snakes [14]. The replacement rate between the functional and first replacement tooth for tooth position d03 of *B*. *constrictor* and position d04 of *P*. *molurus bivittatus* was determined to be 58 days and 93 days, respectively (Table 2).

## Discussion

The teeth of most *Varanus* species (with the exception of *V*. *niloticus* [31]) and their patterns of development and replacement have been only briefly described in the past, with most studies focusing mainly on their feeding behaviour and prey resources. Histological and computed tomography (CT) data presented here provide significant new information about the morphology, patterns of dental development, and tooth replacement that can be directly related to the feeding behaviour of the Komodo dragon, *Varanus komodoensis*. Additionally, the data reveal that the specializations noted in the external and internal dental morphology of the Komodo dragon, in combination with its known feeding behaviour, are ideally suited for predatory behaviour with defleshing as a major component.

Serrations on the tooth crown can be either composed of only enamel, as found in *Smilodon fatalis* [13], or both enamel and dentine cores, known as ziphodonty. Ziphodonty has been previously noted and studied in theropod dinosaurs [12,13,32,33], the sphenacodontid *Dimetrodon* [10], and the mesoeucrocodyle *Mariliasuchus amarali* [34]. Similar to theropod dinosaurs, the serrations are found on both the mesial and distal edges, with the distal exhibiting the denticles throughout the entire crown edge while the mesial only exhibiting them near the apical portion of the crown [35]. We found that *V*. *komodoensis* has true ziphodonty since the histological analysis reveals large dental cores surrounded by enamel for each of the serrations. Among extant varanids and terrestrial vertebrates more broadly, the Komodo dragon appears to be the only predator with large, well-defined dentine cores that contain interdental folds (ampullae) between two adjacent denticles, although previously suggested not to be present in this species [12]. A defleshing behaviour (removing meat sections without swallowing entire prey at once [4]) is primarily the feeding strategy of adult *V*. *komodoensis* with little to no bone-crushing, and we interpret this to be related to the ziphodont type serrations found on both the distal and the mesial crown edges. The serrations on both carinae help with the initial cut of the prey’s soft tissues, while the more numerous and larger-sized serrations on the distal tooth surface enhance the movement of drawing the teeth distally while defleshing prey. The increase in the level of serrations (size and number), including the shift to ziphodonty in adult individuals, may be the reason that larger individuals, typically adults, can deflesh prey in sections [36,37], rather than consuming the prey as a whole [6]. The unique dental characteristic of dentine cores may increase the strength of the serrations to limit the damage imposed on the teeth during feeding for an apex predator [12]. With the addition of hollow, interdental folds, the individual denticles are strengthened since the enamel extends further into the dentine and the denticle has a greater height within the dentine [12]. The morphology of the ziphodonty in the Komodo dragon is distinct from other extinct apex predators, including carnivorous early synapsids and dinosaurs. The serrations are well-defined, with each denticle being slightly spaced apart and having large, external interdental sulci compared to the ziphodonty of theropod dinosaurs [12,32], *Dimetrodon grandis* [10], and *Mesenosaurus efremovi* (T.M. personal observations) which tend to appear smaller and closer together in the labial and lingual view. Additionally, the type of tooth implantation differs between theropod dinosaurs and varanids, with the former having thecodont teeth (teeth which are rooted deep within the alveolus (tooth socket) of the jawbone [38]) [39], whereas the latter has pleurodont teeth (teeth which are attached to the labial wall of the jawbone [38]) [40]. The type of tooth implantation also differs from agamid lizards and chameleons, which have been shown to have mostly acrodont dentition (tooth sits on top of the jawbone, not set within the alveolus [38]), with some pleurodont dentition depending on the location on the jawbone [9,41,42].

Our results have also uncovered a series of other significant correlations between the dentition of the Komodo and its lifestyle. For example, *Varanus komodoensis* is known to experience an ontogenetic shift in habitat and feeding strategy (arboreal to terrestrial [1,6]). Our results illustrate that it simultaneously undergoes a change in tooth morphology. The changes observed in the dentition (premaxillary, maxillary, and dentary) appear to accommodate the different feeding styles found in juveniles and adults. The sharply pointed, labio-lingually compressed teeth found in juveniles represent an adaptation to the available food sources since the small-sized individuals mostly preyed on insects [6]. This simple tooth shape provides a greater chance of capturing food since it allows for easy puncture of small prey, such as insects. Overall, the degree of arboreality appears to be related to size; thus, as the individual *V*. *komodoensis* grows, it gradually shifts its feeding behaviour and habitat to terrestrial, large mammals, with a few instances, the prey being similar in size to the adult Komodo dragon [1,6,43]. The fewer dental specializations observed in juveniles have also been found for *V*. *exanthematicus* with sharply pointed teeth similarly adapted to feeding on small insects, while adults show some interesting heterodonty [21].

Our results indicated that the Indo-Australian varanid clade, including *V*. *komodoensis* and *V*. *salvadorii*, appears to be unique in exhibiting an exceptionally rapid replacement rate, where a new tooth is being formed every 40 days for the Komodo dragon and between 42 to 46 days for the crocodile monitor. In relation to the rapid replacement rate, the functional tooth of the Komodo dragon remains functional only for approximately 92 to 121 days [16]. Additional to the skeletonized data, we have computed tomography data where the replacement teeth have been preserved in position within the soft tissue. This rapid rate of replacement is possible through the maintenance of up to five replacement teeth per functional tooth for the Komodo dragon, and up to four replacement teeth for the crocodile monitor (*V*. *salvadorii*). There appears to be a relationship between the number of replacement teeth produced for a given functional tooth and the rate at which teeth are being replaced. However, *V*. *salvadorii* exhibits only modest dentine cores that are poorly defined and irregularly shaped, which may be related to the different prey sources for *V*. *salvadorii*, primarily feeding on small birds and eggs [1,6]. Interestingly, the replacement pattern differs from the large theropod dinosaurs [12] since the Komodo dragon has rapid replacement rates with multiple replacement teeth per tooth position, while theropods possess longer replacement rates [18]. Interestingly, the replacement rate of the medium-sized Paleozoic hypercarnivorous varanopid, *Mesenosaurus efremovi*, was previously found to be more similar to the Komodo dragon, approximately 35 to 50 days [16].

When we broaden our analysis to some of the other members of the genus, we find that *Varanus salvator* (Asian water monitor) has delicate serrations on both tooth edges and appears to be primarily carnivorous [6]. Although this Asian water monitor normally feeds on larger prey [4,43], the serrations are not well-defined, with very small dentine cores overlayed by thick enamel. Compared to the Komodo dragon, the Asian water monitor produced fewer replacement teeth (up to two) and had slightly longer replacement rates (52 to 59 days). *Varanus exanthematicus* (Savannah monitor), a primarily insectivorous species [6], had a replacement rate (109 days) that was similar to other varanid lizards, such as the Bengal monitor, with a replacement rate of approximately 110 days [14,16]. This typical replacement rate has also been found for green iguanas, *Iguana iguana* [44], as well as in extant *Alligator* and Caiman [15]. Although we did not have the opportunity to sample them histologically, the other two *Varanus* taxa that were examined might have a replacement rate that is within this typical range since only one replacement tooth was observed per tooth position (Fig 6B–6D).

It is clear that patterns of dental development and replacement appear to be related to feeding behaviour. The Komodo dragon is changing its behaviour dramatically during ontogeny in terms of both feeding preference and dental morphology. Ziphodonty is a spectacular feature of the adult Komodo dragon dentition because of its size, and the ontogenetic change in tooth shape appears to be related to the feeding behaviour of *V*. *komodoensis* as an apex predator. Moreover, an apparent reduction in the size of the premaxillary dentition observed in the adult is likely related to the frequent tongue flicking behaviour without needing to depress the lower jaw significantly while foraging for prey. This dental morphology has also been observed in other *Varanus* species studied here and may be a characteristic feature of the genus. *Varanus* appears to be also specialized in tongue morphology, having a slender posterior region and elongated, forked anterior region [45] that is similar to snakes.

The closest extant relative to *Varanus* is the earless monitor lizard, *Lanthanotus borneensis* [46,47], which appears to have a similar anterior tooth morphology, with smaller premaxillary teeth at the midline that increase in tooth height posteriorly. The tongue of *L*. *borneensis* is similar in appearance to *Varanus*, with a slender forked anterior portion [30].

Helodermatidae is one of the closest clades to Varanidae and includes the Gila monster, *Heloderma suspectum*. The Gila monster has a thick fleshy tongue and exhibits a flicking behaviour that functions to detect prey odours [48,49]. The distal end of the tongue is forked, but the forks are short and generally thicker than in varanids [50]. The premaxillary teeth are closer in size (tooth height) to each other and significantly smaller than the rest of the marginal dentition, with the width of the premaxilla matching the width of the tongue. This tooth morphology also allows for a reduced opening between the upper and lower jaws; thus, the lower jaw may be depressed more than in *Varanus* in order to allow the tongue to be released.

*Shinisaurus crocodilurus*, outside of the sister families Varanidae and Lantanotidae, exhibits a different tongue morphology, with a less elongated, weakly forked, and more triangular tongue [30]. *S*. *crocodilurus* has similar-sized premaxillary teeth that are slightly smaller than the maxillary dentition. There is little to no opening between the upper and lower jaw when at rest, and the lower jaw appears to depress more than in *Varanus*, *Lanthanotus*, and *Heloderma*. Snakes have either completely lost or reduced the number of premaxillary teeth, as in *Python molurus bivittatus*, which has two teeth on each side of the premaxillary bone with a gap separating the pairs [51]. As previously noted by Edmund [14] and Gaete and Tucker [52], snakes have multiple generations of replacement teeth, which were now found to have faster replacement rates, such as 86 days for *P*. *molurus bivittatus*.

Interestingly the tongue-flicking in snakes appears to be only used for gathering chemosensory information from the environment [53]. The tongue of varanids is more like those in snakes than in other squamates, and previous research found that members of the clade sampled a larger area of air when performing the flicking action, compared to Helodermatidae and Anguidae, to increase the concentration of chemicals collected [53]. The tips of the widely separated fork on the anterior portion of the tongue allow for a greater surface of air to be sampled from the environment, and the separation of the tips during the flicking action provides additional directional information [54]. Unlike Varanidae and Serpentes, the Iguanidae are considered more generalized lizards and use their tongue for multiple functions, including food manipulation [55].

Our results show a strong correlation between dentition, tongue morphology, and feeding behaviour of the largest known lizard and some of its closest relatives. These anatomical, developmental, and behavioural features will hopefully bring new insights to squamate evolution, including the complex patterns of diversification and convergence among members of this large clade [24,45,55–57]. Additionally, the dental morphology and developmental patterns of the Komodo dragon likely can be used as an analogue for carnivorous early synapsids and dinosaurs to gain a better understanding and reconstruct the feeding behaviours of these extinct taxa. Surprisingly recent phylogenetic analyses using both molecular data and combined morphological and molecular data suggest that varanids are more closely related to Iguania as a whole, rather than snakes [56], indicating that the unique set of exceptional adaptations reported here were acquired independently in the two clades, a startling example of convergent evolution if true.

## Supporting information

S1 Table Morphological measurements for premaxillary teeth. Tooth heights (mm) for all premaxillary teeth and first three maxillary teeth of all *Varanus* and outgroup taxa.
(PDF)

## Abbreviations

ROM: Royal Ontario Museum, Toronto, ON
TNHC: Texas Natural History Collections
FMNH: Field Museum of Natural History, Chicago, IL
UTA: University of Texas
TMM: Texas Memorial Museum
