# Supplementary material for: Exceptionally rapid tooth development and ontogenetic changes in the feeding apparatus of the Komodo dragon
Source: PLoS One. 2024 Feb 7;19(2):e0295002. doi: 10.1371/journal.pone.0295002 (PMC10849390; doi:10.1371/journal.pone.0295002)
Supplement: S1 Table — Tooth heights (mm) for all premaxillary teeth and first three maxillary teeth of all Varanus and outgroup taxa. (PDF) [file pone.0295002.s001.pdf]

## Supporting Information

**S1 Table. Morphological measurements for premaxillary teeth.** Tooth heights (mm) for all premaxillary teeth and first three maxillary teeth of all *Varanus* and outgroup taxa.

| Species | Premaxilla tooth position | | | | | Maxilla tooth position | | |
|---|---|---|---|---|---|---|---|---|
| | 1 | 2 | 3 | 4 | 5 | 1 | 2 | 3 |
| *Varanus komodoensis* (TNHC 95803) | Missing | Missing | 3.13 | 3.45 | 6.29 | 8.59 | 8.92 | 11.89 |
| *Varanus komodoensis* (TNHC 102417) | Replacement | 1.08 | Replacement | 1.13 | 1.39 | Replacement | 2.39 | Replacement |
| *Varanus salvadorii* (ROM R6783) | 1.15 | 0.92 | 1.21 | 1.59 | 2.26 | 3.37 | 4.94 | 5.99 |
| *Varanus salvator* (FMNH 35144) | Missing | Missing | 2.33 | 3.88 | 3.878 | 4.47 | 4.834 | Replacement |
| *Varanus exanthematicus* (FMNH 58299) | 1.11 | 1.37 | 1.41 | 1.69 | 1.853 | 2.44 | 2.221 | 2.57 |
| *Varanus acanthurus* (UTA 13015) | Replacement | 0.65 | Replacement | 0.75 | None | 0.82 | Replacement | 1.09 |
| *Varanus gouldii* (CRF 178) | 1.15 | Missing | 0.56 | 0.61 | 0.888 | Replacement | Replacement | 2.56 |
| *Lanthanotus borneensis* (FMNH 148589) | 0.45 | Replacement | 0.57 | 0.59 | 0.635 | 0.69 | 1.21 | 1.21 |
| *Heloderma suspectum* (TNHC 62766) | 1.69 | 1.78 | 2.04 | 1.96 | None | 2.31 | 3.54 | 3.51 |
| *Shinisaurus crocodilurus* (TNHC 62987) | 0.64 | 0.63 | 0.57 | 0.74 | None | Missing | 1.37 | 1.38 |
